# The visual vernacular: embracing photographs in research

**DOI:** 10.1007/s40037-021-00672-x

**Published:** 2021-06-02

**Authors:** Jennifer Cleland, Anna MacLeod

**Affiliations:** 1 Lee Kong Chian School of Medicine, Nanyang Technological University Singapore, Singapore, Singapore; 2 Division of Medical Education, Dalhousie University, Halifax, NS Canada

**Keywords:** Health professions research, Visual data, Photographs

## Abstract

The increasing use of digital images for communication and interaction in everyday life can give a new lease of life to photographs in research. In contexts where smartphones are ubiquitous and many people are “digital natives”, asking participants to share and engage with photographs aligns with their everyday activities and norms more than textual or analogue approaches to data collection. Thus, it is time to consider fully the opportunities afforded by digital images and photographs for research purposes. This paper joins a long-standing conversation in the social science literature to move beyond the “linguistic imperialism” of text and embrace visual methodologies. Our aim is to explain the photograph as qualitative data and introduce different ways of using still images/photographs for qualitative research purposes in health professions education (HPE) research: photo-documentation, photo-elicitation and photovoice, as well as use of existing images. We discuss the strengths of photographs in research, particularly in participatory research inquiry. We consider ethical and philosophical challenges associated with photography research, specifically issues of power, informed consent, confidentiality, dignity, ambiguity and censorship. We outline approaches to analysing photographs. We propose some applications and opportunities for photographs in HPE, before concluding that using photographs opens up new vistas of research possibilities.

**Supplementary Information:**

The online version of this article (10.1007/s40037-021-00672-x) contains supplementary material, which is available to authorized users.

A Qualitative Space highlights research approaches that push readers and scholars deeper into qualitative methods and methodologies. Contributors to A Qualitative Space may: advance new ideas about qualitative methodologies, methods, and/or techniques; debate current and historical trends in qualitative research; craft and share nuanced reflections on how data collection methods should be revised or modified; reflect on the epistemological bases of qualitative research; or argue that some qualitative practices should end. Share your thoughts on Twitter using the hashtag: #aqualspace

## Introduction

Smartphones, tablets, and other devices are increasingly embedded in everyday life, influencing how many people interact, think, behave and connect with other people [1, 2]. Many people Whatsapp, tweet and text, and/or use Facebook, Instagram and Twitter for professional, social, educational and entertainment purposes. Images are increasingly accessed and used where words would have been used in the past. Indeed, more than 10 years ago, van Dijck [3] suggested that “digital cameras, camera phones, photoblogs and other multipurpose devices are used to promote the use of images as the preferred idiom of a new generation of users.”

The increasing use of digital images for communication and interaction in everyday life has given a new lease of life to a source of research data long embraced by sociology and anthropology [4, 5]. In contexts where smartphones are ubiquitous and with groups of “digital natives” asking participants to share and engage with photographs, this aligns with their everyday activities and norms more than textual or analogue approaches to data collection. Thus, it is time to consider fully the opportunities afforded by digital images and photographs for research purposes [6, 7].

This paper joins a long-standing conversation in the social science literature which advocates moving beyond the “linguistic imperialism” of text [8] to embrace visual methodologies. This conversation has relatively recently made its way into health professions education (HPE): for example, various authors have proposed video [9], video-reflexivity [10] and drawings [11] for research purposes. However, the use of still images or photographs in research remains niche to some areas of inquiry (e.g., exploring patient experiences, particularly mental health and experiences of serious illness (e.g., [12–14]) and some healthcare professions disciplines (e.g., nursing: [15])), and is an under-exploited approach in HPE research (see below for notable exceptions). Yet it is a method which offers many possibilities, particularly in respect to giving research participants more agency and power in the research process than is the case in traditional qualitative data collection approaches such as interviews.

In this paper, we discuss ways of using photographs in research, focusing on the use of photography within participatory research inquiry. We consider ethical and philosophical challenges associated with photography research, as well as its unique strengths. We outline some popular approaches to analysing photographic data. We finish with a brief consideration of how photographs could be used more in HPE research.

## The photograph as data

Photography has been described as a silent voice, another language to communicate with and understand others, and a way of accessing complexities which may not be captured by text or oral language [16]. As instances of Latour’s “immutable, combinable mobiles” [17]—literally things which do not change but which carry action and meaning across time and place, as objects of memory and of relationship—photographs allow us to see what was “happening” at a particular point in time.

Photographs can be a source of data (photo-documentation and existing images) and a tool for eliciting data (photo-elicitation and photovoice). Each of these approaches are explained below.

### Photo-documentation

Photo-documentation has been used in clinical medicine for nearly two centuries [18] Clinical photographs and images are vital for training purposes, to illustrate a clinical finding, steps in a process or procedure, or for comparative (“before” and “after”) purposes. They are an integral part of patient’s clinical notes in numerous specialties and are also used to offer the patient insight into realized or expected treatment results [19].

This way of using photographs—as objective records documenting an objective something—is quite different from how photographs are used in social science research. In fields such as sociology and social anthropology, photography has been used as a tool for the exploration of society [4, 5]. Photographs help others understand how societies are culturally and socially constructed, to critically uncover the meaning people place on certain activities, places, things and rituals and to record and analyse important social events and problems. It is on this second use of photographs in research that we focus from this point onwards.

### Existing images

**Fig. 1:**
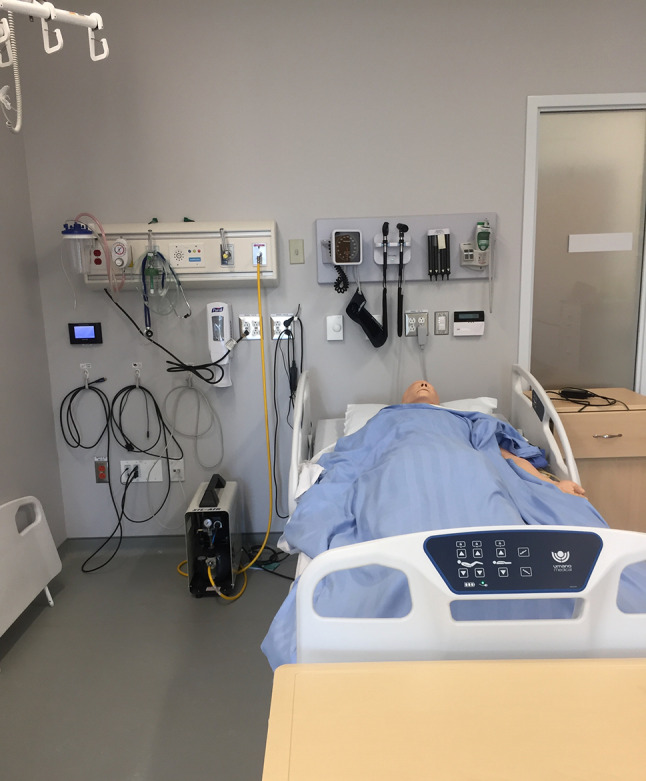
A photograph as an elicitation tool. Collected as part of a sociomaterial study to document the material complexity of simulation led by MacLeod. This photograph of a manikin in a typical simulation suite could serve as a useful elicitation tool in a study of simulation. Rather than asking research participants to use their memories to imagine a simulation suite, the photograph provides concrete detail, helping to reorient participants to the space. Rather than using a phrase like “simulation is complex”, the photograph serves as “evidence” of the complexity, documenting multiple non-human elements involved in a simulation at a particular time and place. This clarity can provide a jumping-off point for more detailed and specific conversations about the topic being studied

A second way of using photographs in research is analysis of publicly available images: in other words, analysis of secondary (photographic) data. There are examples of this approach in medical education in relation to the messages given by images on public-facing documents and webpages, and how these might influence student choice of medical school [20, 21]. Visual data is also used in research examining the relationships between architecture/space and learning [22, 23]. Photographs can show us how people and things relate to each other. For example, what can we learn from a photo illustrating how staff are distributed around a coffee room, or around the table during a morbidity and mortality (M&M) meeting? Documenting the materials of a research space in a photograph serves as a mechanism for tracing the complexity of the field (see Fig. 1 and its accompanying explanation).

### Photo-elicitation

In photo-elicitation (sometimes called photo production [24] or auto-photography), the specific area of focus is typically decided by the researcher. The photos are either taken by the researcher or participants.

In *researcher-driven* photo-elicitation the researcher decides on what people, objects, settings and/or scenes they find interesting or potentially important enough for a picture. These photographs are then used as prompts for discussion within an interview with the researcher. The photo(s) is a prompt to elicit data, akin to an open question in a semi-structured interview. Unlike interview or focus group questions however, participants not only respond to photographs with extended narratives but also supply interpretations of the images, drawing from and reflecting their experiences.

In *participant-driven* photo-elicitation, control of data collection is handed over to participants who have the freedom to pick and choose the representation(s) which is most salient to them in relation to the question under study. For example, to explore children’s experiences of hospital, Adams and colleagues [25] asked children to photograph architectural or design features that most interested them in a vast hospital atrium (the hospital’s primary non-medical space, full of shops, restaurants and so on). The children’s photographs were then used as the anchor to dialogue [26].

Participant-driven photo-elicitation empowers the participant to both choose the image and drive the dialogue about the image. Consider a picture of an alarm clock set to an early hour. This becomes meaningful only when the photographer explains that this image signifies their transition from student to first trained job. While the participant’s perspective on the transition to practice could potentially be accessed using traditional words-alone methodologies, photographs are different because they present what the participant felt was worthy to record, help capture the immediacy of the experience and stimulate memories and feelings. In other words, one of photo-elicitation’s strengths, and how it differs from interviews and focus groups, is its potential to collect data that not only taps into the perspective of the participant but does so at the time of the experience.

Images seem to prompt a different kind of reflection on lived experiences. Harper [26] suggests that images prompt emotions and thoughts in ways that narrative alone cannot. By seeing what they did, informants may help the researcher to better understand their behaviour. Moreover, by viewing and discussing photos together, the researcher and participant actively co-construct meaning. In this way photo-elicitation offers a way to potentially enrich and extend existing interview methodologies and give a combination of visual and verbal data for analysis purposes [27]. Furthermore, the act of interpreting an image creates a critically reflective space within the research process which is lacking in interview methods. Leibenberg suggests that “collectively then, images introduced into narrative research create important links that participants can use to more critically reflect on their lived experiences and to more accurately discuss and share these experiences with others” [28, p. 4].

Arguably however, if the main source of data is not the photographs themselves but the transcripts from photo-elicited discussions, this may still privilege participants who are more skilled verbally—maintaining the “linguistic imperialism” of text, or, more accurately, of transcribed responses [8]. While this criticism cannot be wholly dismissed, the many empirical studies referred to in this paper suggest that photographs help make abstract ideas accessible and encourage reflection in groups which are less literate and who do not routinely engage in reflection. Moreover, there are approaches to data analysis which privilege the image, not the accompanying text (see below).

### Photovoice

A specific research method within the bracket of photo-elicitation is photovoice. Developed by Wang and Buriss in 1997, photovoice involves asking community members to identify and represent their community through specific photographs and tell the stories of what these pictures mean, promoting critical dialogue and potentially catalysing social action and change [29]. Photovoice allows people to see the viewpoint of the people who live the lives, and as such is considered an example of participatory research [30]. For example, MacLeod et al. [31] asked adolescent youth to take photographs pertaining to the health of their community. The adolescents created a platform for discussion, and helped the researchers, who were medical students, learn about the community they were serving. Photovoice is often used to access and explore patient experiences, particularly mental health and experiences of serious and/or life-threatening illness [12–14].

The ease of taking photographs with a mobile phone has opened up new ways to utilise the photovoice methodology, particularly the method of “time-space diaries” [32] or digital journals. Participants record what is meaningful to them across time and activities, such as what and where they ate over a full day, or salient events in the first few weeks of medical school. Just like non-research social media activity, a series of images can provide insight into real, lived experiences and give participants a voice to reflect on their everyday lives on issues relevant to the research topic. Consider a resident taking pictures of things and people who were significant to their first experience of a full weekend shift. The nature of the images may change over time, reflecting exposure to different patients, working with different colleagues, task demands and fatigue.

In summary, the nature of photographs as data varies according to who produces them, whether they are independent of the research or created specifically for the research, how they are used in the research process and whether they are used in conjunction with narrative (verbal) data. These key decisions can be synthesised, according to Epstein et al. [33], into three basic questions:

- Who is going to make or select the images to be used in the interviews?
- What is the content of the images going to be?
- Where are the images going to be used, and how?

How photographs and accompanying narratives can be analysed is discussed next.

## Data analysis

There are two main ways of approaching photographic data analysis. The first, the dialogic approach, focuses on analysing the verbal or written reflection on the content of photographs and what they symbolise. This approach is fundamentally constructivist, “locating visual meaning as foundational in the social construction of reality” [34, p. 492]. Traditional ways of analysing verbal/transcribed data such as thematic analysis [35], content analysis [36], grounded theory [37] and various forms of discourse analyses [38] are appropriate for analysis of photograph-prompted dialogue. In this approach, the photographs themselves are usually used merely for illustration purposes, if at all [38].

Alternatively, the data can be within the photograph itself, separate from its capacity to generate dialogue and independent of any explanation. Photographs can provide new ways of seeing the phenomena under study from their visual features. For example, in their analysis of existing images on medical school websites and prospectuses in 2019, MacArthur, Eaton and Marrick [21] recorded information including gender, ethnicity, assumed role and setting, of each person on each image. They found a predominance of hospital-themed images, compared to few community-themed images. They concluded that these images signalled to students a strong preference for hospital-based settings, despite a strong national drive to recruit more general practitioners.

This approach to analysis is referred to as “archaeological” because images inherently reflect the social norms of a point in time. Consider your graduating class photograph. Clothes and hairstyles which were chic at the time may look old-fashioned and incongruous when viewed many years later. In this way, photographs contain “sedimented social knowledge” [34, p. 502], manifest through the photographer’s choices of scenes, subjects, styles, compositions and so on. An educational example of this is presented in Photograph S1, found in the Electronic Supplementary Material (ESM).

Grounded, visual pattern analysis (GVPA) combines both approaches [39]. Via a structured, multi-step process of analysis, GVPA investigates the meanings individual photographs have for their photographers and also attends to the broader field (sample) level meanings interpreted from analysis of collections of photographs. Paying attention to absence (what is not photographed) is also important [40]. The analysis process ends by building conceptual contributions rather than purely empirical ones from the photographic data (see Photograph S2 in ESM).

Whichever analysis approach is taken, as with any qualitative research, it is important to consider quality and rigour in respect to the credibility, dependability, confirmability and transferability of the data [41]. Providing details of the sampling strategy, the depth and volume of data, and the analytical steps taken helps ensure credibility and transferability. Photo-elicitation allows participants to work with and direct the researcher to generate data that is salient to them, thereby increasing the confirmability of research outcomes. Allowing participants to clarify what they meant to convey in their photographs is inherently a form of member checking. As for all research, ethical considerations should be considered and addressed, as well as a clear statement made on formal research ethics committee approval or waiver. Thought must be given to the power relationship between researcher and participants and how this might affect recruitment, the nature of the data and so on. Reflexivity, reflecting on the extent to which similarities or differences between researcher and researched may have influenced the process of research, is particularly critical in photo-elicitation studies [42]. Keeping written field and methodological notes as well as a reflexive diary is important for dependability and confirmability.

Finally, in terms of data presentation, in our discipline most journals have a limit on the number of tables, figures and/or images allowed per paper, and most do not publish colour photographs. This limits the visual data which can be presented in an article. However, journals also offer the option of supplementary e‑files. We suggest that one or two pictures in an article can support key evidential points, with additional data made available electronically.

## Ethical considerations associated with photographs in research

As with any method, care must be taken to ensure the proper use of photographs for research purposes. Here we briefly consider the main ethical issues of power, informed consent, anonymity, dignity and image manipulation. We direct readers to Langmann and Pick [43] for more in-depth discussion.

### Power

In any researcher/participant situation, there is a power dynamic that privileges the “expert” researcher over the object of study, the participant. Certain ways of using photographs in research, specifically photo-elicitation, can change this dynamic and empower participants by giving them an active role in the research process, making them the experts, and allowing the researcher greater insight into participant perspectives [29, 30]. Photo-elicitation also gives those who are not verbally fluent another way to express themselves effectively, avoids the use of survey questionnaires and other research instruments that might be culturally biased, and places participants as equals—able to reflect and decide how they want to represent themselves visually [43]. Photo-elicitation is thus firmly rooted in an approach to inquiry that draws on Paulo Freire’s (1970) critical pedagogy [44] and fits within the broader participatory action research method [29, 30, 37–40, 45].

The use of mobile phones for data collection is considered a way of connecting younger groups with research [46], connecting with populations in more remote and rural communities across the globe [47] and with “difficult to reach” populations (e.g., [14]). However, it is important to again acknowledge the “digital divide” and the associated power differential: marginalised populations and certain societal groups may not have access to equipment to take and share photographs. Where this is the case, the researcher must consider whether to supply the necessary equipment or whether an alternative method of data collection is more feasible.

### Informed consent

Informed consent is particularly challenging with photographs. It is difficult to ensure that every person in an image has given their consent to the photo being taken and used for research purposes. Where images are participant-generated, clear instructions about the purpose of the research and the photographs, and the processes of ethical consent, are essential [48].

### Confidentiality

Confidentiality is an issue, particularly if a photograph includes a person’s face. Faces can be pixelated or blurred to protect participants’ identities, but these approaches may objectify the people in the photo and make the photographs less powerful [48].

### Dignity

Our third point relates to dignity. Langmann and Pick [43] suggest three ways of considering dignity in research photography: being sensitive to the social and cultural norms of the communities being researched, being aware that those who are the focus of the research will benefit by the presentation of an authentic view of the situation and considering the impression the photograph will give if and when it is published. In all cases, it is the researcher’s responsibility to exclude photographs which are not covered by ethical approvals, as well as any potentially harmful or compromising photographs.

### Ambiguity

Photographs can mean different things to different people [24, 49] and meanings may change over time, depending on context and how they are associated in terms of text and other images (for example, one’s interpretation of a photograph taken as a teenager is likely to differ when viewing it as an adult). This ambiguity makes some researchers uncomfortable. However, if one takes a social constructivist stance, that we live in a multi-reality world, then this possibility of multiple meanings from a photograph adds to the data.

### Censorship

Conscious and unconscious “self-censorship”, including when, where or what to photograph, or editing a photo to convey an intended message, is inherent in photo-elicitation [45]. However, self-censorship is not an issue if one accepts that the purpose of photo-elicitation is to access the social reality of another individual.

## Strengths of using photographs in research

### Participation and co-construction

As mentioned earlier, photo-elicitation and photovoice maximise opportunities for participant agency and engagement in the research process, allowing participants to work with and direct the researcher. Furthermore, in dialogic approaches, research involves a joint process of knowledge-production where narratives are co-constructed between participant and researcher through discussion. By using participant-driven photographs, the researcher gains an understanding of what the content of the photos means to the participants without imposing their own framework or perception of a topic on the process.

### Trust

Participatory research requires trust, a safe space between participant and researcher, so people can express their thoughts and views. Wicks and Reason [50] suggest that establishing this safe space must be considered throughout the research process: empowering participants in the earlier stage of the research process can also build the connection and trust between researcher and participant—and reduce participant inhibition later on. This may be particularly useful where the topic is sensitive or taboo. For example, Meo [51] reported photo-elicitation was useful in tapping “class and gendered practices” (p. 152) in greater depth than with interviews alone.

Giving power to participants within the research process can be challenging for researchers. Adjusting to participants as co-researchers may be new and unfamiliar. Continuous flexibility and reflexivity on a personal (e.g., personal assumptions, values, experiences, etc. that shape the research) and epistemological (e.g., the limits of the research that are determined by the research question, methodology and method of analysis) considerations are critical [52].

### Structure

Photographs provide structure to an interview, giving the researcher something to return to, to elicit more detailed discussions and/or trigger memories and reflection [53]. In addition, participants often give information about people or things outside of the photo (the invisible) as well as on who and what are visible [52]. Similarly, the researcher may be able to access parts of participants’ lives that would be difficult to see into otherwise. For example, Bourdieu argues that visual methods of research may be particularly helpful in investigating habitus, ways of being, acting and operating in the social environment that are “beyond the grasp of consciousness” [54, 55].

### Snapshots in time and of space

**Fig. 2:**
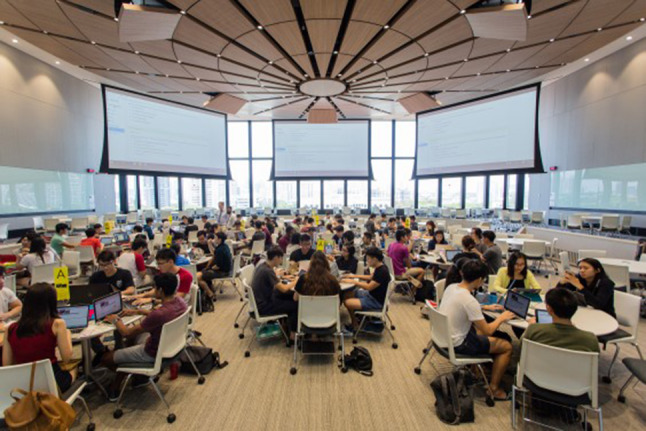
An example of a photograph representing the assemblage of time and space: Students distributed in the space of a contemporary learning suite. Photograph from a publicly facing webpage on a medical school website. This photograph provides an example of how a photograph captures space and time. It provides a glimpse at a contemporary medical school. The photograph serves to document the complexity of modern medical schools, making clear the digitized learning environment. Such a photograph might evoke emotion and a sense of progress, in particular, when contrasted with more traditional images of students learning in a stadium-style lecture theatre

As mentioned earlier, photographs are inherently snapshots in time. They also provide snapshots of space, a means of examining the material assemblages of space, of how things are ordered and used [56]. For example, a photo of students in a learning space would illustrate who sits with whom, the spatial relationships between humans (e.g., student and student, students and teachers) and the non-human (e.g., bags, laptops, phones, snacks) (see Fig. 2 as an example).

## Applications and opportunities

Looking forward, we encourage researchers to consider the use of photographs as a source of data, as a way of accessing data that might otherwise have been obscured or overlooked if we had relied solely on language-based data. We encourage readers to consider what might be learned were we to augment current understanding by incorporating photographic data sources into healthcare professions research. In Table S1, found in ESM, we suggest some outstanding research questions and topics that could be explored. The list found there is by no means exhaustive. Rather it reflects our own interests and observations and should be regarded as a springboard to help readers consider diverse ways in which photographs may add richness in research endeavours.

## Conclusion

There are many ways of conducting qualitative research in health professions education research (HPER). All have their affordances and limitations. In this article, we have offered a critical examination of how photographs can be used to generate rich and potentially different data to that generated through talk-only data collection. Using photographs in HPER research opens up new vistas of research possibilities, whether as a means of accessing snapshots of people and situations in time and space and/or as a means of engaging participants collaboratively, to explore taken-for-granted lived experiences which may not otherwise be accessible. This is a fertile area for future research and the empirical potential is vast, ranging from reflective practice to widening participation to questions which are as yet unknown.

## Supplementary Information

Table S1 Potential applications and opportunities for using photographs in qualitative HPE research. This is arranged by area of Interest (e.g., simulation), potential research question, philosophical underpinnings, methodology, method and analysis for ease

An archeological example of the complexity of distributed medical education. Taken in the audio-visual control room of a video-conferenced medical education program (from MacLeod’s photograph research cannon)

This example features a photograph from a publicly facing webpage on a medical school website. The combination of the photograph and its accompanying text would lend itself well to a Grounded Visual Pattern Analysis (GVPA)
