# Supplementary figures and images for: The visual vernacular: embracing photographs in research
Source: Perspect Med Educ. 2021 Jun 2;10(4):230–7. doi: 10.1007/s40037-021-00672-x (PMC8368779; doi:10.1007/s40037-021-00672-x)

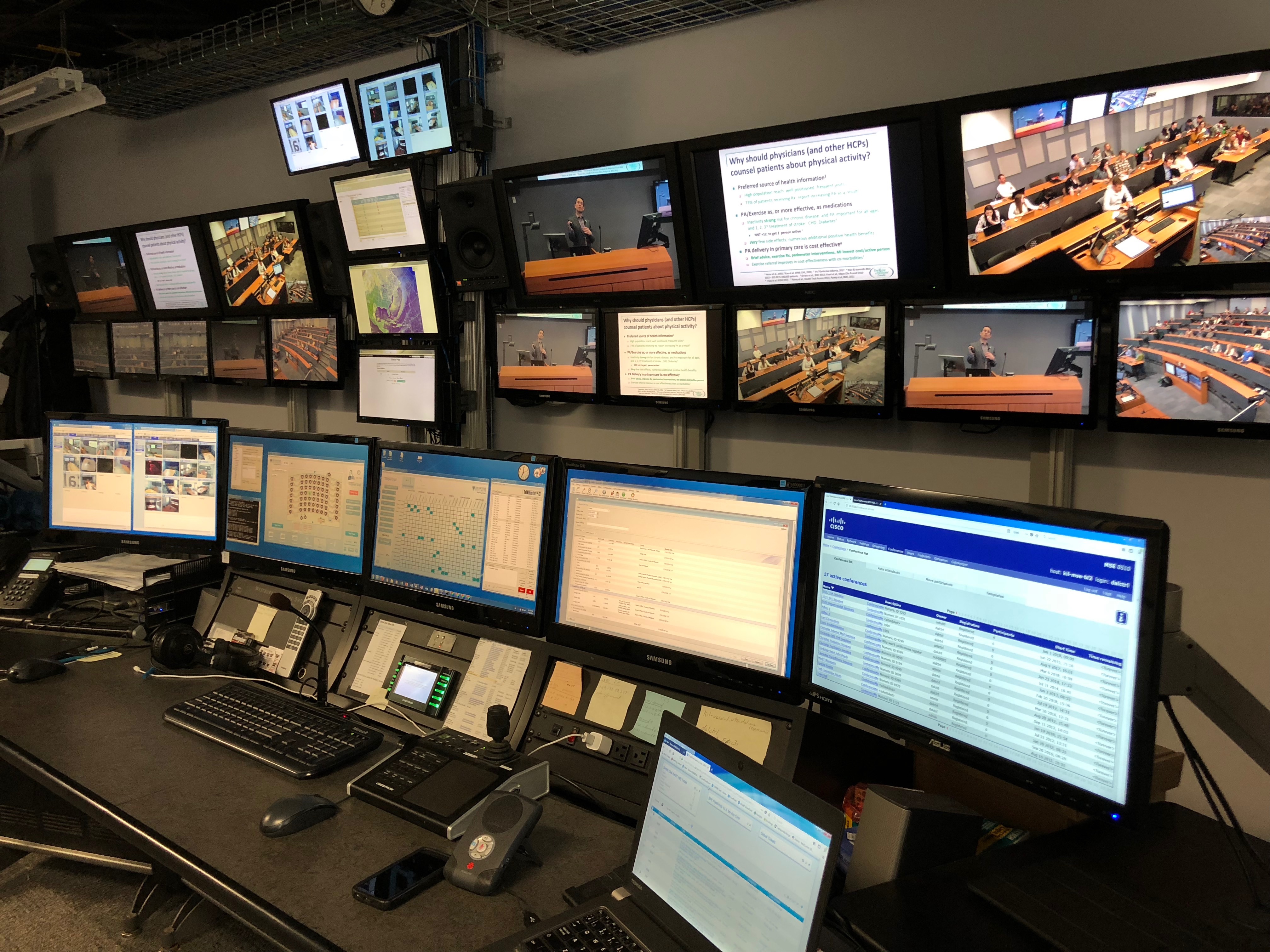

Supplement: Supplementary file 1 — Table S1 Potential applications and opportunities for using photographs in qualitative HPE research. This is arranged by area of Interest (e.g., simulation), potential research question, philosophical underpinnings, methodology, method and analysis for ease [file 40037_2021_672_MOESM1_ESM.jpg]

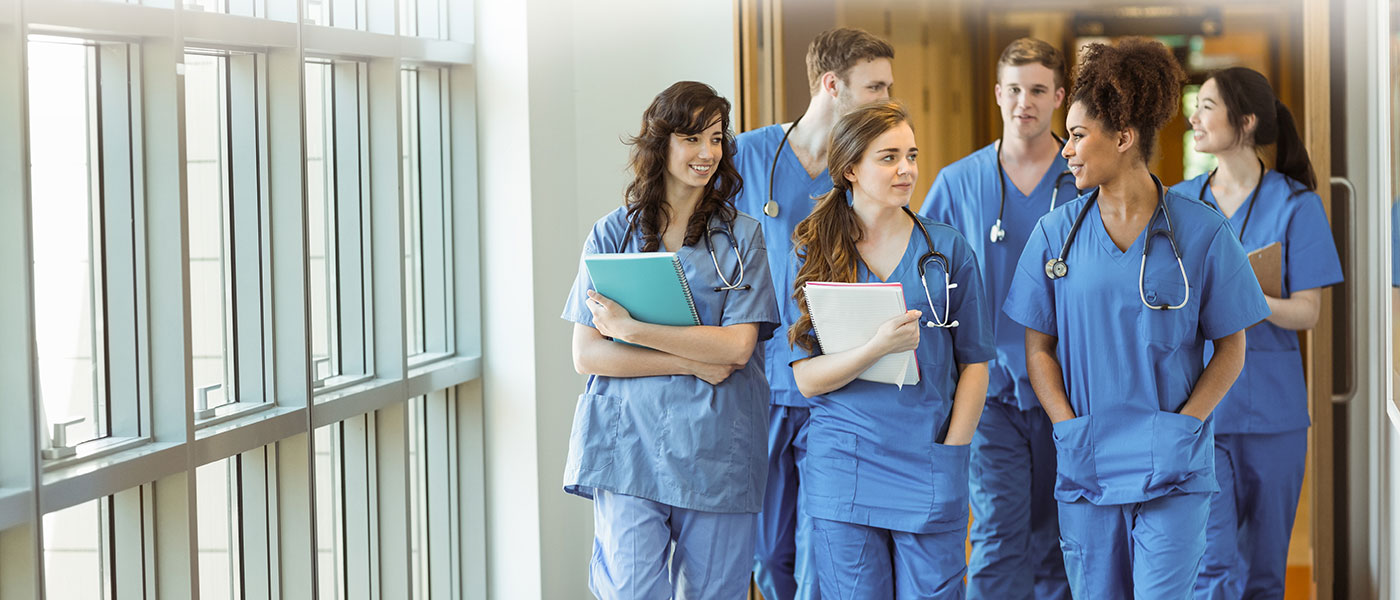

Supplement: Supplementary file 2 — An archeological example of the complexity of distributed medical education. Taken in the audio-visual control room of a video-conferenced medical education program (from MacLeod’s photograph research cannon) [file 40037_2021_672_MOESM2_ESM.jpg]
