# Supplementary material for: The visual vernacular: embracing photographs in research
Source: Perspect Med Educ. 2021 Jun 2;10(4):230–7. doi: 10.1007/s40037-021-00672-x (PMC8368779; doi:10.1007/s40037-021-00672-x)
Supplement: Supplementary file 3 — This example features a photograph from a publicly facing webpage on a medical school website. The combination of the photograph and its accompanying text would lend itself well to a Grounded Visual Pattern Analysis (GVPA) [file 40037_2021_672_MOESM3_ESM.docx]

**Table S1** Potential applications and opportunities for using photographs in qualitative HPE research

| **Area of Interest** | **Potential Research Question** | **Philosophical underpinnings** | **Methodology** | **Method & Description** | **Analysis** |
| --- | --- | --- | --- | --- | --- |
| Clinical learning environments | How does the built environment influence learner engagement? | Constructionist | Case study | Photo-documentation  Researchers take photographs of a variety of spaces in which formal medical education curriculum is delivered, with a specific focus on capturing a diversity of learning environments | Grounded, visual pattern analysis focusing on interpretation of context |
| Community engagement | How do community members perceive their healthcare challenges? | Action research | Participatory action research | Photovoice  Community-based research participants are provided with cameras, or use their own devices, to document their experiences of healthcare challenges in their respective communities | Grounded, visual pattern analysis focusing on understanding challenges from the perspectives of participants |
| Learner mistreatment | How do trainees experience mistreatment in the learning environment? | Critical theory | Autoethnography | “Selfie” style photographs  A researcher uses their personal device to document their physical and emotional reactions to incidents of mistreatment | Dialogic, analysis of the significance associated with each photograph |
| Microaggressions in medical education | What are the everyday experiences of students from underrepresented groups in medical school? | Social constructionism | Participatory action research | Visual storytelling  Research participants might use their devices to capture examples of microaggressions they experience (for example, focusing on gender binaries, a researcher might take photos of forms that make assumptions about gender, lavatory signage, ideas about clothing, etc.) | Grounded, visual pattern analysis, focusing on piecing together various photographs to tell a story about microaggressions |
| Procedural skills teaching | How do learners’ suturing skills evolve over time? | Realism | Case study | Photographic timeline  A researcher might take a series of photographs of students sutures, focusing on tracing progress | Archaeological, tracing the development of a skill over time |
| Reflective practice | How do clinicians experience the phenomenon of making an error? | Constructivist | Phenomenology | Photograph as a tool for guiding reflection  A researcher provides a photograph representing a clinical encounter that did not go well, and asks participants to reflect on why the encounter was not successful, what they might do differently, and why | Grounded, visual pattern analysis, focusing on reconnecting participant voice with structured viewing and theorizing based on the photographs |
| Teaching and learning | How do students conceptualize good teaching? | Critical pedagogy | Participatory action research | Photo-elicitation  Photograph of a “good” teaching scenario as a tool for inspiring in-depth conversation about what constitutes good teaching | Grounded, visual pattern analysis, focusing on how people conceptualize strong teaching skill |
| Technologically enabled learning | How do the technologies of videoconferencing influence participation in an online lecture? | Sociomaterial | Case study | Photo as material transcript  Photographs serve as a record of the material elements in the scenario (screens, discussion boards, cameras, etc.) providing a visual voice to the non-human actors | Archaeological, focusing on tracing of material affordances documented in photographs |
| Widening participation/increasing diversity in medical education | What is the lived experience of under-represented minority (URM) medical students? | Freire’s critical pedagogy | Phenomenology | Photovoice  Participants capture photographs of their challenges and barriers during medical education, which inform interviews | Dialogic – analysis of the narratives around participant photographs |
